# Visualization of Small Vessels by Micro–Computed Tomography Using Titanium Dioxide Nanoparticles as a Novel Contrast Agent

**DOI:** 10.1155/ijbi/6688558

**Published:** 2025-01-30

**Authors:** Taku Goto, Ruriko Tanabe, Hirotoshi Shibuya, Masaru Tamura, Shintaro Nomura

**Affiliations:** ^1^Faculty of Bioscience, Nagahama Institute of Bio-Science and Technology, Nagahama, Shiga, Japan; ^2^Mouse Phenotype Analysis Division, RIKEN BioResource Research Center, Tsukuba, Ibaraki, Japan

## Abstract

Angiography by means of micro–computed tomography (m-CT) is extensively used for the diagnosis of vasculature disorders. To establish a connection between m-CT images and genuine histopathology findings, we developed two novel titanium dioxide nanoparticle (TiO_2_-NP)–based perfusion contrast agents: TiNpCA-1 and TiNpCA-2. Three-dimensionally reconstructed m-CT images in mice perfused with these contrast agents showed high resolution and accuracy in various organs without deformation or dilation of vessels. Vessels < 20*  μ*m in diameter were clearly visualized by m-CT, and capillaries of 4 *μ*m in diameter were visualized by nano-CT. After perfusion, the contrast agents were kept in the vessels by the formation of an aggregate with ethanol. Histological samples were prepared from CT-scanned specimens. No perfusion-induced damage or abnormal structures were observed. The signals of the contrast agents were detected clearly, and the tissue histology was of adequate quality for pathological diagnosis. Agglomerates of TiO_2_-NPs were present in both agents; their approximate sizes were 1.0 and 6.0 *μ*m in TiNpCA-1 and 1.5 *μ*m in TiNpCA-2. We considered that these agglomerates were trapped within capillaries at the beginning of perfusion. And at the end of perfusion, vessels of larger size were filled with agglomerates. These findings suggest a direct correlation between the signal intensity in m-CT imaging and the volume of contrast agent entering the vessels, indicating a quantitative aspect to the system. The low *K*-edge value of titanium (4.6 KeV) ensures that the signal intensity of the contrast agent remains unaffected at low energies (40 KeV). Lower energy levels improve the contrast-to-noise ratio. Consequently, using titanium dioxide as a contrast agent allows us to achieve a higher contrast-to-noise ratio while maintaining a favorable signal-to-noise ratio. Our results strongly support the notion that TiO_2_-NPs as a contrast agents hold promise not only for investigating circulatory disorders in experimental pathology but also for uncovering new insights in anatomical physiology.

## 1. Introduction

To address the increasing number of patients with circulatory disorders, researchers are conducting preclinical studies, particularly those involving genetically modified small animal models. The goal of these studies is to provide valuable information for diagnosis, treatment, and development of novel medicines. Abnormal vascular structures are mainly detected using computed tomography (CT) and histology. Recent improvements in the resolution of micro–computed tomography (m-CT) and nano–computed tomography CT (n-CT) now allow for detailed imaging of blood vessel structures with a resolution of better than 10 *μ*m [1]. Despite these improvements, however, consistent visualization of blood vessels < 30*  μ*m in diameter remains challenging [2, 3]. To understand the physiological and pathological features of living organisms, it is crucial to achieve clear visualization without altering the size and shape of structures. This is particularly important because many arterioles, arteriovenous anastomoses, and capillaries in experimental small animals fall within this size range (< 30 *μ*m in diameter). Terminal arterioles (10 *μ*m in diameter) contain elastic tissues, smooth muscle, fibrous tissue, and endothelium. However, capillaries (4–8 *μ*m in diameter) that are linked to terminal arterioles contain only endothelium [4]. The smooth muscle in the arterioles plays a crucial role in regulating the internal space of the vessel, thereby maintaining blood pressure at a specific level. Consequently, the adjustment of blood flow quantity and direction into capillaries or arteriovenous anastomoses is influenced by the arteriole's smooth muscle [5]. Examination of changes in arterioles is important because various diseases affect the size and shape of arterioles, including diabetes [6], autoimmune disease, chronic obstructive pulmonary disease, and inflammation [7]. The lack of a biomedical imaging system with which to explore small vessels may cause serious delays in the investigation of various disorders.

Selecting an appropriate vascular contrast agent is important to obtain m-CT images with a high signal-to-noise ratio and contrast-to-noise ratio. Even in postmortem studies, several commercially available preclinical agents such as iodine-, barium-, lead-, and gold-based contrast agents have provided unsatisfactory images of small vessels. While preparing samples, these contrast agents easily flow out of tissues. Moreover, most of them damage the cell morphology, resulting in poor histologic images. Precise diagnosis of lesions may require not only angiography but also histology. An optimal contrast agent should cause minimal damage or artificial change to the structure and histology of the vasculature; have an affinity for vessels, remaining in them permanently at a high concentration; and demonstrate signal intensity that correlates with the physiological blood flow rate, facilitating quantitative analysis of the results.

In the present study, we focused on a series of nanoparticles (NPs) large enough to remain within the vessels. Among a variety of commercially available NP solutions that we have tested, an aqueous solution of titanium dioxide nanoparticles (TiO_2_-NPs) did not cause any histological changes to the morphology of the tissues. Paraffin sections made from titanium dioxide (TiO_2_)-perfused tissues were stained with hematoxylin–eosin, immunohistochemical stains, and other special stains such as periodic acid–Schiff. Based on the results, we decided to use the TiO_2_-NP solution as the contrast agent for angiography in mice.

TiO_2_-NPs have a high refractive index (*n* = 2.4), which makes them useful for various applications including medicines, coatings, inks, plastics, food, cosmetics, and textiles. The manufacture of TiO_2_-NPs involves the use of sol–gel, flame hydrolysis, coprecipitation, impregnation, and chemical vapor deposition processes [8]. A very important property of TiO_2_-NPs is their ability to undergo steric stabilization, which increases the stability of the particles in biological systems. This property also allows for their use in drug delivery systems because they can conjugate with anticancer drugs or antibodies specific to cancer cells [9]. The photocatalytic properties of TiO_2_-NPs are also useful for the development of medications. The large surface area of TiO_2_-NPs provides a large reaction area, which results in good photocatalytic activity. And the bandgap structure found in TiO_2_-NPs absorbs ultraviolet radiation. These properties are used in both photodynamic therapy [10] and sonodynamic therapy [11].

TiO_2_-NPs are also used in imaging diagnosis. Iqbal et al. [12] reported that iron absorbed in TiO_2_-NPs showed contrast enhancement in magnetic resonance imaging and improved the photodynamic therapeutic efficacy of TiO_2_. Braz et al. [13] reported effective multimodal dental imaging using TiO_2_-coated fluoride NPs. Moreover, the effect of silica/gold nanoshells and TiO_2_-NPs on the optical properties of the skin was demonstrated by Kirillin et al. using optical coherence tomography [14]. To the best of our knowledge, however, successful use of TiO_2_-NPs in CT imaging has not been reported to date. This is likely because titanium does not belong to a high atomic number group, which makes it challenging to generate an intense signal in an x-ray image.

Commercially available TiO_2_-NP solutions contain dispersion agents to prevent agglomerate formation. In many cases, compounds containing ester structures are used as dispersion agents [15]. By changing the ionic strength, pH, temperature, and the concentration of organic solvents, agglomerates are formed in TiO_2_-NP solutions. And the size of agglomerates is known predictable in some extent [16–19]. This work explores the use of such agglomerates as contrast agents for m-CT. Finally, this report describes the development of TiO_2_-NP-based contrast agents for m-CT, providing a novel method for integrating bioimaging and histopathology.

## 2. Materials and Methods

### 2.1. Animals

Six- to 8-week-old ICR female mice (25–30 g) were purchased from CREA Co. Ltd. (Tokyo, Japan). The mice were housed in a temperature-controlled high-barrier facility (Nagahama Institute for Bioscience and Biotechnology) with free access to mouse chow and water and kept under a 12-h light/dark cycle. All procedures were performed in accordance with the guidelines for the Care and Use of Laboratory Animals and all other relevant guidelines and regulations of the Animal Research Committee of Nagahama Institute for Bioscience and Biotechnology; the same institute approved the study (Approval No. 2023–80).

### 2.2. Chemicals

All chemicals were purchased from FUJIFILM Wako Chemicals Co. Ltd. (Osaka, Japan) unless otherwise noted.

### 2.3. TiO_2_-NP Solution

Commercially available TiO_2_-NP solutions were obtained from Tayca Co. Ltd. (Okayama, Japan). The crystal form of TiO_2_ is anatase. According to the manufacturer's website, the solvent for WD0456, TKS-203, and TKD-801 is water and the average primary particle size is 100–400 nm for WD0456 and 6 nm for TKS-203 and TKD-801 (https://www.tayca.co.jp/english/research_development/new_tech01/nanodispersions/spec.php).

Microphotography was conducted using a 100× oil-immersed objective lens which was documented with a DP27 camera (Olympus, Tokyo, Japan). The particle size in TiO_2_-NP solutions was estimated using 1 *μ*m (standard deviation < 0.05*  μ*m) and 5 *μ*m (standard deviation < 0.5*  μ*m) of “microparticle size standard based on polystyrene monodisperse” (Sigma–Aldrich St. Louis, Missouri, United States) as a size-standard marker.

### 2.4. Preparation and Physical Properties of Contrast Agents

**Figure 1:**
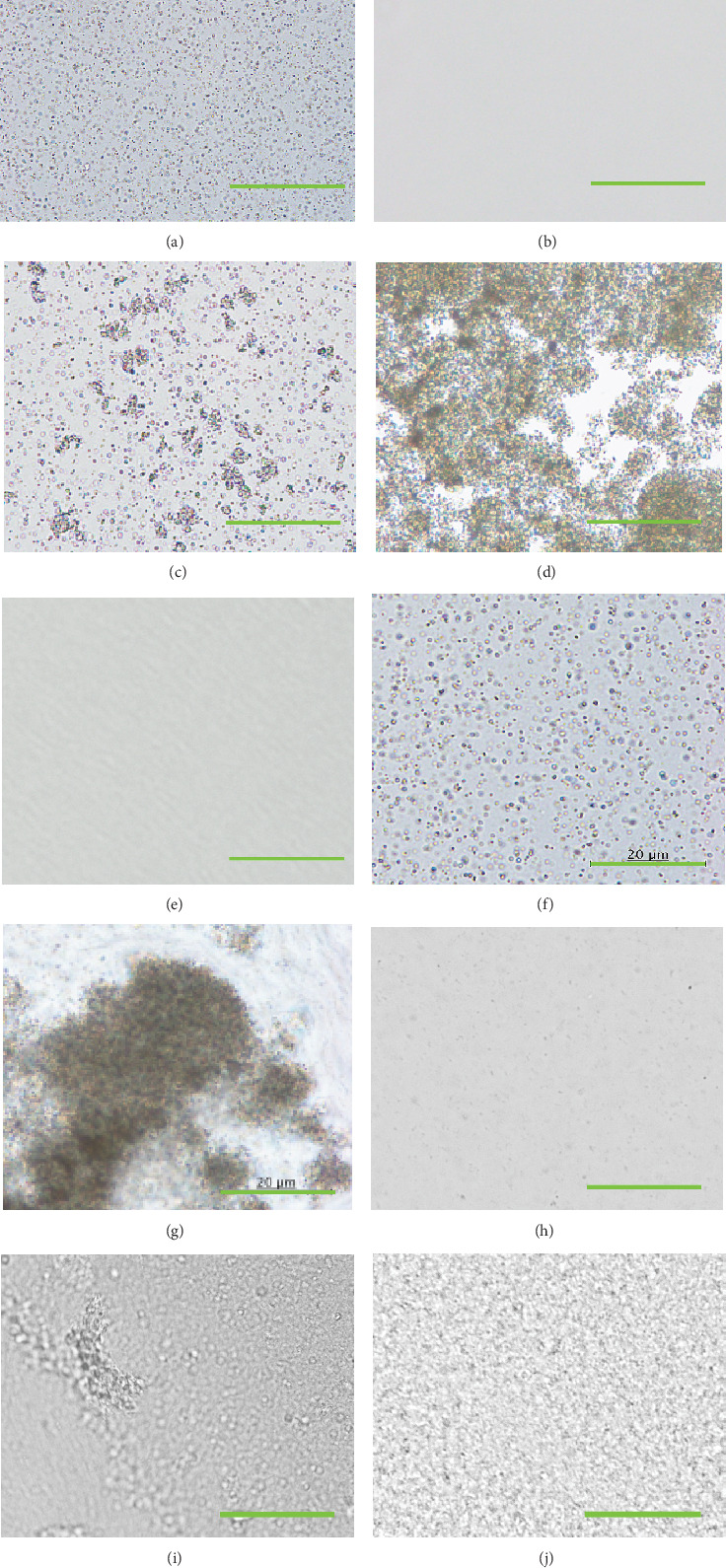
Changes in the sizes of TiO_2_ particles in aqueous solutions and their mixtures. To prepare TiNpCA-1, (a) WD0456 and (b) TKS-203 were mixed. (c) Microparticles of 6 and 1.5 *μ*m in size were detected after 5 min. (e) To prepare TiNpCA-2, TKD-801 was mixed with phosphate-buffered saline. (f) Microparticles of 1 *μ*m in size were detected after 5 min. Heavy aggregation in (d) TiNpCA-1 and (g) TiNpCA-2 was observed in the presence of 40% ethanol. Various sizes of agglomerates were detected in (h) WD0456, (i) TKS-203, and (j) TKD-801 in the presence of 70% ethanol, but no heavy aggregation was observed in (h), (i), and (j). Bar = 20*  μ*m.

TiO_2_-NPs of WD0456 were barely observable by microscopy at high magnification (Figure 1(a)). The estimated size of the particles was < 0.5 *μ*m, corresponding to the manufacturer's datasheet (100–400 nm). The average primary sizes of the TKS-203 and TKD-801 particles were 6 nm in the manufacturer's datasheet and difficult to detect (Figures 1(b) and 1(e)). TiNpCA-1 is a 2:1 mixture of two aqueous TiO_2_-NP solutions (WD0456 and TKS-203). After 5 min of the preparation of TiNpCA-1, two types of polygonal-shaped agglomerates were observed. The size of the smaller one is approximately 1.0 *μ*m, and the size of the larger one is approximately 6.0 *μ*m (Figure 1(c)). The size and population of each agglomerate showed no significant change until 60 min after preparation. TiNpCA-2 is a 2:1 mixture of aqueous TiO_2_-NP solution TKD-801 and phosphate-buffered saline (PBS) (Nacalai Tesque Inc., Kyoto, Japan). Five minutes after preparation of TiNpCA-2, polygonal-shaped particles of approximately 1.5 *μ*m were observed (Figure 1(f)). And the size and population of agglomerates showed no significant change until 60 min after preparation. Angiographical images using TiNpCA-1 and TiNpCA-2 as contrast agents showed good reproducibility within 60 min of preparation. The physical properties of TiNpCA-1 and TiNpCA-2 were also examined. The density and viscosity of TiNpCA-1 were 1.12 g/mL and 3.33 mPa·s, respectively, and those of TiNpCA-2 were 1.20 g/mL and 3.52 mPa·s, respectively. These values are comparable to those of whole blood [20]. After adding ethanol at 40%, heavy aggregation was observed in TiNpCA-1 and TiNpCA-2 (Figures 1(d) and 1(g)). Various sizes of agglomerates were detected in (h) WD0456, (i) TKS-203, and (j) TKD-801 in the presence of 70% ethanol, but no heavy aggregation was observed in (h), (i), and (j).

### 2.5. Perfusion of Contrast Agents

The mice were deeply anesthetized by peritoneal injection of 200 mg/kg secobarbital (Nichi-Iko Pharmaceutical Co. Ltd., Toyama, Japan), and the heart was exposed via intercostal incision. A 25-G butterfly catheter was inserted into the left ventricle, parallel to the septum, and 25 mL 0.9% saline solution was perfused from the left ventricle and drained from the clipped right atrium until the drainage color turned clear. Then, the mice were fixed by perfusing 15 mL neutralized formalin at a flow rate of 5 mL/min. Next, 3–6 mL TiNpCA-1 or TiNpCA-2 was perfused 5 min after their preparation as contrast agents. The viscosity of the contrast agents was measured with a tuning-fork vibration viscometer (SV-1A; A&D Co. Ltd., Tokyo, Japan). To control the hydrostatic pressure of the contrast agents against the wall of the left ventricle within the range of 90–135 mmHg, the bag filled with TiNpCA-1 was suspended 109–163 cm above the mice, resulting in a flow rate of 4.0–5.3 mL/min, and the bag filled with TiNpCA-2 was suspended 102–153 cm above the mice, resulting in a flow rate of 4.2–5.5 mL/min. The hydrostatic pressure and the amount of TiNpCA-1 used were dependent on the target organ. For the kidney, 2.0 mL TiNpCA-1 was perfused at 104 mmHg. For the brain and retina, 5.0 mL TiNpCA-1 was perfused at 135 mmHg. For the liver, 5.0 mL TiNpCA-2 was perfused at 135 mmHg.

### 2.6. Preparation of Samples for CT Analysis and Histology

After perfusion of the contrast agent, incisions were made in the abdomen and skull. And the mice were immersed in 70% ethanol overnight. Then, appropriate perfusion was confirmed by conventional m-CT. The mice were dissected, and the desired tissues were dehydrated in an ethanol series, cleared with xylene, and embedded in paraffin at its melting point of 56°C–58°C (Merck Millipore Corp. Massachusetts, United States). The paraffin block of embedded samples was analyzed by high-resolution m-CT or n-CT. Paraffin sections of 4-*μ*m thickness were cut, deparaffinized, hydrated, and stained with hematoxylin and eosin for histological analysis.

### 2.7. CT Imaging

Conventional scanning was performed with an m-CT system (m_CT2; Rigaku Corporation, Tokyo, Japan) at 50 kVp, 180 mA, and 512 views. Detailed scanning was performed using an x-ray m-CT system (ScanXmate-E090S; Comscantecno Co. Ltd., Yokohama, Japan) at 40 kVp, 100 mA, and 1000 views. Finally, limited areas such as the kidney cortex were scanned with 8.0-KeV x-rays generated by a copper target (40 kV, 30 mA; spot size of 70 *μ*m; no filter), capturing a total of 700 projections using an n-CT system (nano3DX; Rigaku Corporation). To visualize the hepatic sinusoid structure, the liver was scanned for 4 h by with 8.0-KeV x-rays generated by a copper target (50 kV, 80 mA; spot size of 5 mm; no filter). Three-dimensional (3D) reconstruction of CT images was carried out using an OsiriX DICOM viewer (Pixmeo Sàrl Co. Ltd., Geneva, Switzerland).

## 3. Results

### 3.1. CT Scanning of Perfused Samples by the Contrast Agent TiNpCA-1

After perfusion of PBS and formalin, WD0456, TKS-203, or TiNpCA-1 was injected from the left ventricle with careful maintenance of the hydrostatic pressure within the physiological range to avoid dilation of vessels. When WD0456 or TKS-203 was used for perfusion, TiO_2_ readily flowed out from the right atrium, and only faint signals were detected by m-CT (data not shown). However, when TiNpCA-1 was used, distinct signals were detected in variously sized arteries (6–1000 *μ*m). After perfusion of TiNpCA-1, incisions were made in the abdomen and skull. And the mice were immersed in 70% ethanol overnight. Then, the mice were dissected; the organs and tissues were removed, dehydrated, embedded in paraffin, and scanned by m-CT. When organs and tissues were removed in prior to ethanol treatment, intensities of signal in vessels were decreased. This means that ethanol treatment is not only for dehydration but effective to retain TiO_2_ in the vessels presumably forming heavy aggregation of contrast agent.

**Figure 2:**
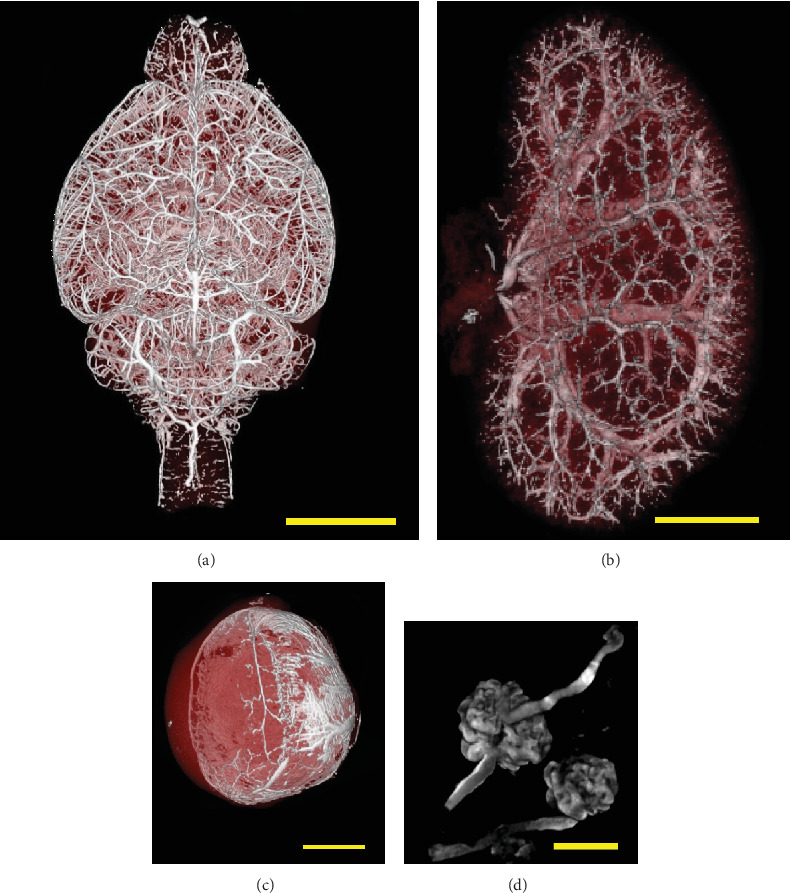
Three-dimensionally reconstructed images of blood vessels in ICR mice perfused with TiNpCA-1 shown in CT scans. (a) Brain (bar = 5 mm), (b) kidney (bar = 3 mm), and (c) eyeball (bar = 0.5 mm). (d) Microvessels in the glomerulus perfused with TiNp-1 were visualized via n-CT (nano3DX; Rigaku Corporation, bar = 50*  μ*m).

The scanning protocol for the embedded specimens was experimentally designed to optimize the signal-to-noise ratio. To discern the spatial localization of lesions necessary for pathological analysis, a 3D reconstructed image produced by the OsiriX software served as a reference. Distinct signals were obtained from various organs including the brain, kidney, and eyeball (Figures 2(a), 2(b), and 2(c)). In addition, microstructures such as the blood vessels of the renal glomerulus were clearly visualized (Figure 2(d)). Strong signals from the contrast agent were detected in blood vessels > 20*  μ*m within various organs such as the lung, heart, adrenal gland, ovary, uterus, testis, and thyroid. No obvious abnormal structures such as dilation, blind ends, or aneurysms were detected in the reconstructed images of the organs examined. Horizontally rotated 3D reconstructed images of the brain, kidney, and retina were documented in videos (Supporting Information 2: Data V1, V2, and V3, respectively). These showed few abnormal structures compared to previous reports and could be used for the diagnosis of bleeding, blood clots, infarction, and aneurysms in small animals. The hydrostatic pressure of the contrast agent being perfused is important when using this method; it should be kept in the physiological range of 90–135 mmHg [21]. Although it is possible to perfuse saline, formalin, and contrast agents using a tube pump under controlled flow rates within the range of 4.0–5.3 mL/min, caution should be exercised to account for potential artifacts in the results, such as vessel dilation and corruption.

### 3.2. Histology of CT-Scanned Samples

**Figure 3:**
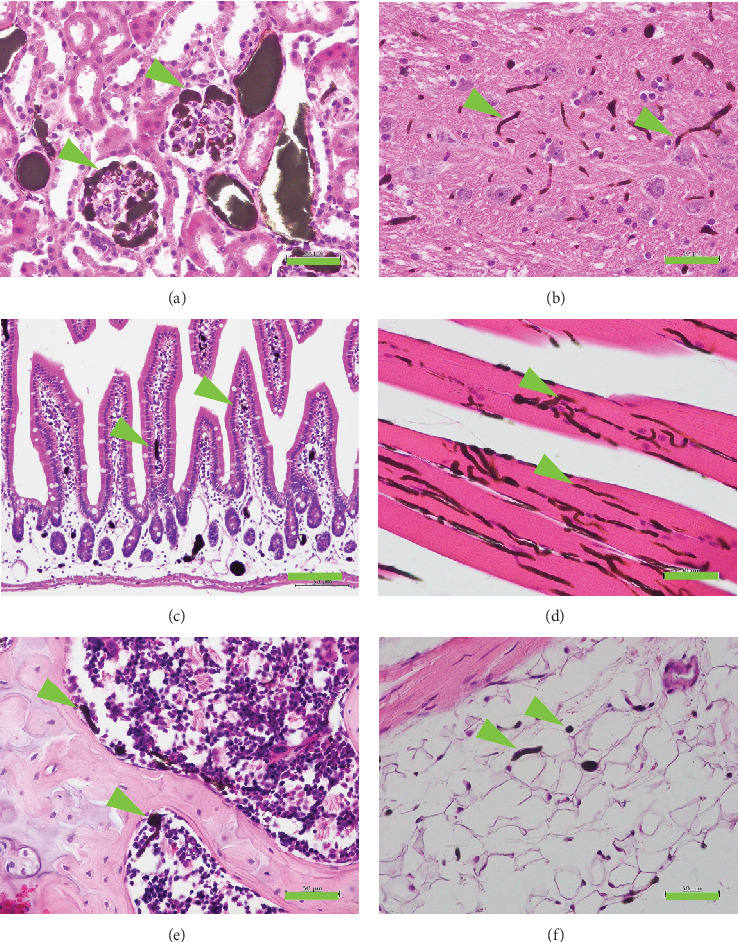
Detection of contrast agent in histological sections. Perfused specimens were dehydrated and embedded in paraffin. The TiNpCA-1 signals were black (green arrowheads). Sections of 4-*μ*m thickness were stained with hematoxylin and eosin. Histology of the (a) kidney, (b) cerebrum, (c) intestine, (d) skeletal muscle, (e) bone marrow, and (f) dermis is shown. Bar = 50*  μ*m.

TiNpCA-1 signals were also detected histologically in all vessels > 20*  μ*m and in a considerable proportion of vessels < 10*  μ*m (Figure 3). The presence of TiNpCA-1 was detected as a dark green to black color signal in microvessels of the glomerulus (6-*μ*m diameter) (Figure 3(a)), cerebral cortex (6-*μ*m diameter) (Figure 3(b)), intestine (5-*μ*m diameter) (Figure 3(c)), skeletal muscle (4-*μ*m diameter) (Figure 3(d)), bone marrow (5-*μ*m diameter) (Figure 3(e)), and dermis (4-*μ*m diameter) (Figure 3(f)). The quality of the histological results was adequate for precise pathological diagnosis.

### 3.3. Visualization of Sinusoidal Vessels in the Liver by TiNpCA-2

**Figure 4:**
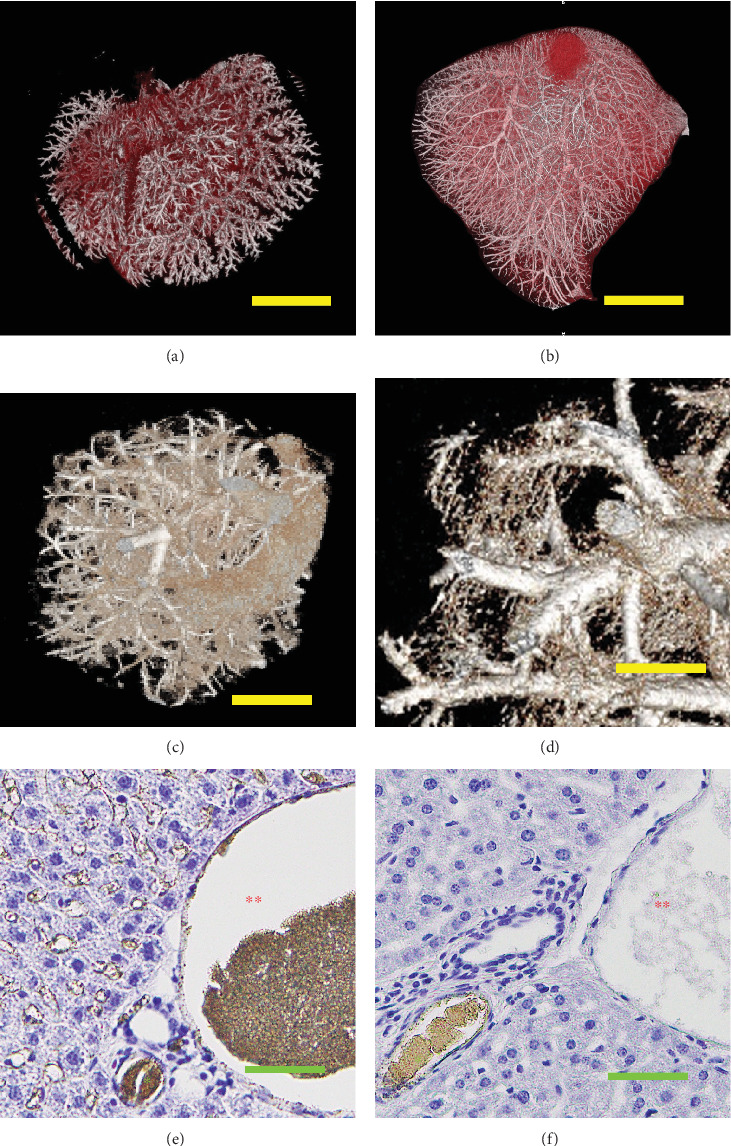
Three-dimensionally reconstructed CT images and histology of the livers of ICR mice perfused with (a, f) TiNpCA-1 and (b–e) TiNpCA-2. Livers were dissected from perfused mice, embedded in paraffin, and scanned with (a, b) ScanXmate-E090S (Comscantecno Co. Ltd., Yokohama, Japan) or (c, d) n-CT system (nano3DX; Rigaku Corporation). Paraffin sections were prepared and stained with hematoxylin (e, f). (e, f) A hepatic artery (green asterisk) and portal vein (red asterisk) are shown. (e) Contrast agent was detected on the surfaces of sinusoid structures (green arrowheads). Bars = (a, b) 10 mm, (c) 500, (d) 200, and (e, f) 50 *μ*m.

Although TiNpCA-1 was also detected in the liver, almost all of the signals were located in hepatic arteries (Figure 4(a)). Histological analysis revealed signals in hepatic arteries but not in the portal vein (Figure 4(f)). The portal vein blood flowed through the gastrointestinal tract, spleen, gallbladder, and pancreas. Thus, before entering the liver, the blood passed microvessels of these organs once. We assumed that agglomerates of TiNpCA-1 were trapped in the capillaries of these organs before entering the portal vein. Therefore, we developed another contrast agent (TiNpCA-2) which did not contain larger agglomerates (6 *μ*m) found in TiNpCA-1 (compare Figure 1(c) with Figure 1(f)). When TiNpCA-2 was used, strong signals were detected not only in the hepatic arteries but also in the portal vein (compare Figure 4(b) with Figure 4(e)). Furthermore, in contrast to TiNpCA-1, the signal of TiNpCA-2 was detected in the sinusoids of the liver (compare Figure 4(e) with Figure 4(f)), where blood flowed toward the central vein. TiNpCA-2 was detected on the surfaces of sinusoid structures (green arrowheads). Reconstructed 3D images of the perfused liver indicated that the organ was filled with TiNpCA-2 flowing from the portal vein to the sinusoids and microvessels of the liver (compare Figure 4(e) with Figure 4(d)). When only TKD-801 with PBS was used as the aqueous TiO_2_-NP solution for perfusion, TiO_2_ readily flowed out of the right atrium, and only faint signals were detected by m-CT (data not shown).

## 4. Discussion

We constructed a diagnostic system for models of circulatory disorders that combines m-CT and histology and uses novel contrast agents that incorporate TiO_2_-NPs.  Figure 1 shows how the sizes of NPs change during the process of preparing the contrast agents. The mechanisms by which the different sizes of agglomerates are formed in our TiNpCA-1 and TiNpCA-2 formulations are not fully understood. However, several potential mechanisms are suggested as follows.

WD0456 is a dispersed TiO_2_-NP of which surface is coated with aluminum (Al). And TKS-203 is an aqueous solution of titanium phosphate. Therefore, the surface of WD0456 is positively charged and the surface of TKS-203 is negatively charged in neutral pH. When they were mixed, they were supposed to interact each other and form agglomerates. Other possibility is that the disperse agent in WD0456 caused competitive absorption to TKS-203 and resulted in the instability of dispersion in WD0456. However, it is still difficult to explain the size of agglomerates formed in TiNpCA-1 at present. After preparation of TiNpCA-1, larger (6 *μ*m in size) and smaller (1.0 *μ*m in size) agglomerates showed rapid increase within 5 min, and no distinct change was observed until 60 min. So, it is possible to speculate that the different mechanisms lie in the process of larger and smaller agglomerate formation. Polyacrylic acid is used as a dispersion agent in TKD-801; when TKD-801 was mixed with PBS, sodium (Na) ion derived from PBS was absorbed in the dispersion agent which possibly causes the shortage of dispersion effect, resulting the formation of agglomerates in TiNpCA-2. Heavy aggregations were observed in ethanol-treated TiNpCA-1 and TiNpCA-2 but not in WD0456, TKS-203, and TKD-801. Dispersion states of TiNpCA-1 and TiNpCA-2 are supposed to be uneven and be unstable compared to WD0456, TKS-203, and TKD-801. Once unstable, TiNpCA-1 and TiNpCA-2 may form agglomerates, as previously reported [22], where the stability and agglomeration of alumina NPs in ethanol–water mixtures were discussed [22]. Although the exact mechanisms remain elusive, our formulations have demonstrated high reproducibility in producing m-CT images.

Regarding the technical details of the imaging technique (resolution and artifacts), commonly used silicone rubber–based contrast agents, such as Microfil, are highly viscous and require pressure far in excess of blood pressure. As a result, they are prone to vessel dilation, leakage, and occlusion and can produce artificial structures that differ from the in vivo situation (Supporting Information 1: Figure S1 (a–c)). This is a major problem when performing angiography in experimental animals. Therefore, to minimize changes in vessel structure, we ensured that the density and viscosity of the contrast agents matched those of whole blood. We were also careful to maintain the hydrostatic pressure of the contrast agents during perfusion within the physiological range. Consequently, detailed angiograms of the arteries were successfully obtained for various organs and tissues (Figure 2). This means that the new contrast agent we developed can be delivered into the capillaries at the same pressure as blood, which sufficiently reduces the risk of vasodilation or dilation due to perfusion pressure (Supporting Information 1: Figure S1 (d–f)). Reduced contrast agent clogging also contributes to improved resolution of capillary imaging. On the other hand, the signal intensities in small vessels within digestive organs, including the stomach, intestine, and colon, were inconsistent despite robust signals in vessels > 50*  μ*m in such organs. To investigate this, we prepared samples without formalin perfusion before the injection of the contrast agent, yielding results comparable to those with formalin perfusion. Our findings imply that the signal intensities correlated with the rate of blood flow into specific organs, modulated by arteriolar control. Efforts to clarify this mechanism should be continued. In this case, a report that blood distribution patterns throughout the body can be physiologically affected [5] would also be considered. Consequently, this system holds the potential for quantitative analysis.

Histological analysis revealed the presence of TiNpCA-1 in capillaries with a diameter of 4–6 *μ*m. The effective retention of contrast agents within the vessels may be attributable to the high concentration of ethanol during the dehydration process. As noted in the Results section, the removal of organs and tissues prior to ethanol treatment led to a reduction in signal intensities within the vessels. Therefore, further analysis of the effects of ethanol (and other solvents) on the dispersion of TiO_2_-NPs should be conducted to improve formulation.

Notably, the shape and morphology of the vessels appeared normal, and the vessels were filled with contrast agents (Figure 3). When a smaller amount of contrast agent (2 mL) was used for perfusion, strong signals were observed predominantly in the capillaries and small vessels. Based on these results, we propose that contrast agents may have accumulated in the vessels as follows. Agglomerates of 1.0 *μ*m became trapped in the capillaries, whereas those of 6.0 *μ*m were captured in small arterioles, “stacking up” as if the vessels were a filtering apparatus. Ultimately, the vessels became saturated with contrast agents, resulting in signals of high intensity.

The sinusoids in the liver were effectively visualized with TiNpCA-2 as the contrast agent but not with TiNpCA-1 (Figure 4). When TiNpCA-2 was used for perfusion, small vessels and capillaries were visualized and a faint signal was observed within the veins. However, a strong signal was seen in the sinusoids and portal vein. The reason for the different localizations of TiNpCA-1 and TiNpCA-2 is not clear at present. However, different to TiNpCA-1, TiNpCA-2 was detected on the surfaces of sinusoid structures. So, it is possible to discuss the particular localization of TiNpCA-1 and TiNpCA-2 may have been associated with differences in the solvents used and/or the affinity of the contrast agents to tissues. We counted the number of particles in each agent and estimated that 20%–30% of the NPs in TiNpCA-1 turned to smaller (1.0 *μ*m) agglomerates and that 10%–20% f of the NPs in TiNpCA-1 turned to larger (6 *μ*m) agglomerates. Similarly, 20%–30% of NPs turned to 1.5-*μ*m agglomerates in TiNpCA-2. Anyhow, the precise mechanisms responsible for the differential localization and distribution of the two contrast agents remain to be elucidated.

It is not clear why TiO_2_-NPs made satisfactory results compared to other contrast agents. The possible cause of agglomeration is that Na ion derived from PBS was absorbed to dispersion agent possibly causing the shortage of dispersion effect, resulting in the formation of agglomerates in TiNpCA-2.

Because CT examinations are typically performed within an energy range of 80–140 kV, materials containing atoms with *K*‐edge values > 30 KeV such as iodine, barium, erbium, gold, and lead are often used. Among these agents, erbium-based NPs have been successfully employed in mouse angiography [23]. Therefore, we tested erbium-based NPs. Although we obtained a strong signal in the kidney, the images of other organs were unstable. To avoid agglomeration of agents, it is necessary to perform sonication and decantation multiple times before using them; otherwise, they may readily adhere to the vessels, leading to dilation. The signal intensities of the atoms commonly used in contrast agents have maximum values in their specific ranges, which are higher than those of soft and hard tissues. In contrast, because of the low *K*-edge of titanium, a position of maximum peak lies between soft and hard tissues. For pathological diagnosis, it is important to know the spatial localization of imaged vessels with surrounding tissue. Therefore, titanium may be useful for pathological examination [23–25]. Despite the fact that titanium has a lower *K*-edge (approximately 4.9 KeV [26]), which is typically considered below the expected range for a contrast agent, it is detectable at 40 KeV. Consequently, high-contrast images similar to those obtained in low-energy CT have been achieved [27]. As a result, the peripheral blood vessels of small animals become easier to detect.

TiO_2_-NPs have been used in a variety of products for various purposes. However, they have not been widely applied in angiography [28, 29]. TiNpCA-1 and TiNpCA-2 can be easily prepared by mixing commercially available and quality-controlled products. In the laboratory, we first prepared the contrast agents and confirmed the formation of agglomerates by microscopy. After perfusion of saline and formalin, the agents were perfused. The desired organs or tissues were removed from the mice which had been immersed in 70% alcohol, and conventional m-CT was used to determine whether the perfusion was complete. Paraffin-embedded specimens were scanned using high-resolution m-CT. We examined the structures of vasculature by carefully analyzing the images and determining the precise positions and orientations of lesion(s). We aligned the positions of lesions based on 3D images and generated histological sections. Perfusion was completed in < 10 min utilizing only 6 mL contrast agent for each mouse. Furthermore, the histological detection of contrast agent highlights the value of this system for the diagnosis of pathologies.

Finally, it is important to note the necessity of considering both the broader applications and limitations of TiO_2_-NPs. A comprehensive analysis of potential limitations, such as toxicity or biocompatibility concerns in vivo, is crucial, especially when considering clinical translation. The investigation of in vivo toxicity and biocompatibility remains a significant challenge that warrants further study in the future.

The contrast agents developed in this study are not only cost-effective but also timesaving, potentially paving the way for numerous advancements in bioimaging and histopathology.

## 5. Conclusion

Angiography using the novel contrast agents described herein holds promise as a valuable tool for investigating the environmental and genetic causes of circulatory disorders in small vessels. Moreover, the use of these contrast agents may contribute valuable insight into the field of anatomical physiology in the future.

## Data Availability

Materials, data, and associated protocols are available from corresponding author on request.

## Supporting Information

Supporting Information 1 Figure S1: Comparison of angiography image with silicone rubber contrast agent and TiNpCA-1. Maximum intensity projection (MIP) (a) and 2D stacked (b) images of the brain, and MIP image of the kidney (c) using a conventional silicone rubber as contrast agent for angiography. It is difficult to visualize microvessels with conventional silicone rubber agent due to its high viscosity. The high viscosity of the contrast agent causes artificial structural changes in angiography, such as contrast agent occlusion and vessel dilation. MIP (d) and 2D stacked (e) images of the brain and MIP image of the kidney (f) using TiNpCA-1. Angiography with TiNpCA-1 shows uniform perfusion throughout the brain and kidney and does not show any artifacts such as vessel dilation. Scale bar = 1 mm.

Supporting Information 2 Videos V1–V3: Horizontally rotated 3D reconstructed images of the brain, kidney, and retina. Data V1: Video V1, brain. Data V2: Video V2, kidney. Data V3: Video V3, retina.
