# Supplementary figures and images for: Visualization of Small Vessels by Micro–Computed Tomography Using Titanium Dioxide Nanoparticles as a Novel Contrast Agent
Source: Int J Biomed Imaging. 2025 Jan 30;2025:6688558. doi: 10.1155/ijbi/6688558 (PMC11824794; doi:10.1155/ijbi/6688558)

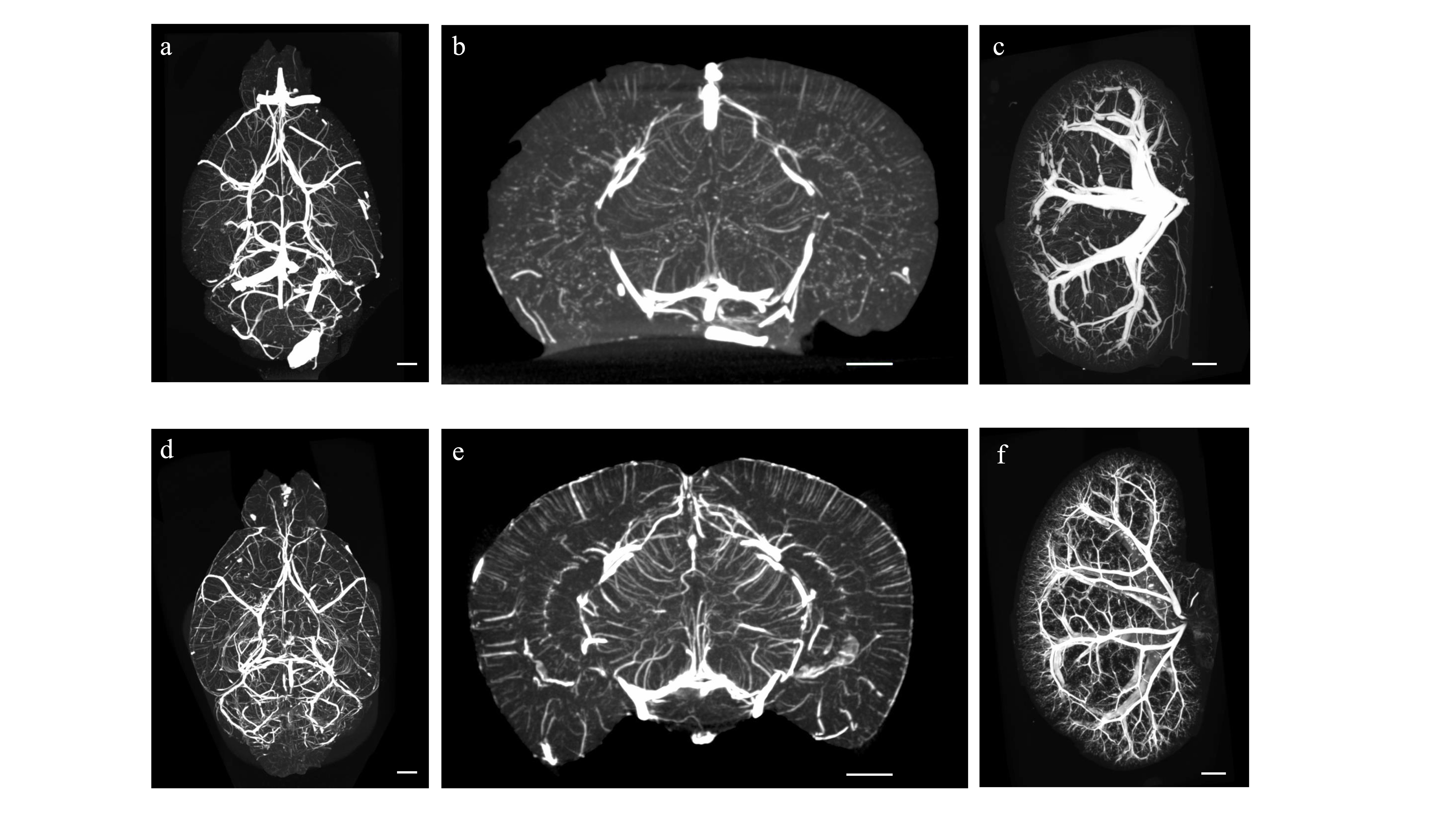

Supplement: Supporting Information 1 — Figure S1: Comparison of angiography image with silicone rubber contrast agent and TiNpCA-1. Maximum intensity projection (MIP) (a) and 2D stacked (b) images of the brain, and MIP image of the kidney (c) using a conventional silicone rubber as contrast agent for angiography. It is difficult to visualize microvessels with conventional silicone rubber agent due to its high viscosity. The high viscosity of the contrast agent causes artificial structural changes in angiography, such as contrast agent occlusion and vessel dilation. MIP (d) and 2D stacked (e) images of the brain and MIP image of the kidney (f) using TiNpCA-1. Angiography with TiNpCA-1 shows uniform perfusion throughout the brain and kidney and does not show any artifacts such as vessel dilation. Scale bar = 1 mm. [file 6688558.f1.jpg]
